# Meaning in Life of Aging Family Caregivers in Home Hospice Care

**DOI:** 10.1093/geroni/igaf122.2615

**Published:** 2025-12-31

**Authors:** Haerim Lee, Hyun-E Yeom, Eunyoung Park, Misook Jung

**Affiliations:** Chungnam National University, Daejeon, Republic of Korea; Chungnam National University, Daejeon, Republic of Korea; Chungnam National University, Daejeon, Republic of Korea; Chungnam National University, Daejeon, Republic of Korea

**Keywords:** meaning in Life, Aging, Home Care Services, Hospices, End-of-Life, Caregivers, Qualitative Research

## Abstract

Families caring for home hospice patients directly witness end-of-life experiences, which, alongside their aging process, may lead to a reevaluation of life’s meaning. As they navigate the complexities of caregiving and observe the end-of-life journey firsthand, their perceptions of life’s meaning may become increasingly intricate and uncertain. This study explores how family caregivers of home hospice patients perceive and interpret life’s meaning throughout the caregiving process. This qualitative phenomenological study employed face-to-face, semi-structured interviews with nine family caregivers (M age = 67 years) who cared for hospice patients at home. Data were analyzed using Colaizzi’s method, leading to the identification of three key themes: (1) diminished sense of life’s meaning amid physical and emotional exhaustion, (2) uncertainty and disorientation in meaning-making following bereavement, and (3) the challenging pursuit of life’s meaning amid suffering. Family caregivers experienced vulnerability due to the demands of 24-hour caregiving and the physical limitations associated with aging, making it difficult to find meaning in life. They also struggled with uncertainty in redefining their lives without the patient while simultaneously grappling with an ambivalent sense of responsibility in seeking a renewed sense of purpose. These findings highlight the complex interplay between caregiving dedication, personal sacrifice, and the ongoing reconstruction of life’s meaning throughout the caregiving journey. They underscore the critical need for tailored psychosocial support to assist family caregivers in navigating the challenges of post-caregiving life and age-related difficulties.

